# Osteogenic and Angiogenic Profiles of Mandibular Bone-Forming Cells

**DOI:** 10.3389/fphys.2019.00124

**Published:** 2019-02-19

**Authors:** Barbora Veselá, Eva Švandová, Jan Bobek, Hervé Lesot, Eva Matalová

**Affiliations:** ^1^Laboratory of Molecular Morphogenesis, Institute of Animal Physiology and Genetics, Czech Academy of Sciences, Brno, Czechia; ^2^Department of Physiology, University of Veterinary and Pharmaceutical Sciences, Brno, Czechia

**Keywords:** mandibular bone, intramembranous ossification, angiogenesis, osteogenesis, PCR Array

## Abstract

The mandible is a tooth-bearing structure involving one of the most prominent bones of the facial region. Mesenchymal cell condensation is the first morphological sign of osteogenesis, and several studies have focused on this stage also in the mandible. Little information is available about the early post-condensation period, during which avascular soft condensation turns into vascularized bone, and all three major bone cell types, osteoblasts, osteocytes, and osteoclasts, differentiate. In the mouse first lower molar region, the post-condensation period corresponds to the prenatal days 13–15. If during this critical period, when osteogenesis reaches the point of major bone cell differentiation, vascularization already has to play a critical role, one should be able to show molecular changes which support both types of cellular events. The aim of the present report was to follow in organ context the expression of major osteogenic and angiogenic markers and identify those that are up- or downregulated during this period. To this end, PCR Array was applied covering molecules involved in osteoblastic cell proliferation, commitment or differentiation, extracellular matrix (ECM) deposition, mineralisation, osteocyte maturation, angiogenesis, osteoclastic differentiation, and initial bone remodeling. From 161 analyzed osteogenic and angiogenic factors, the expression of 37 was altered when comparing the condensation stage with the bone stage. The results presented here provide a molecular survey of the early post-condensation stage of mandibular/alveolar bone development which has not yet been investigated *in vivo*.

## Introduction

The mandible is a tooth-bearing structure and thus necessary particularly for mastication but also for speech, aesthetical appearance, and human well-being. The mandibular bone represents an attractive target for tissue engineering and regenerative approaches (Ward et al., 2010). An essential prerequisite of such approaches is an understanding of mandibular osteogenesis *in vivo*, including the accurate description of molecular signalisation associated with sequential, and possibly distinct, developmental steps. Among these, the osteogenic and angiogenic processes dominate (Grosso et al., 2017), and notably, they often involve same molecules such as CD36 (Simantov and Silverstein, 2003; Kevorkova et al., 2013), Sphk1 (Pederson et al., 2008), and Vegfa (Zelzer et al., 2002).

The mandibular bone, apart from the condylar process, is intramembranous and its development can be divided into a pre-osteogenic phase, which covers mesenchymal condensation, and later post-condensation stages including the majority of osteogenic differentiation. Several studies have focused on the pre/condensation events (e.g., Jabalee et al., 2013; Kaul et al., 2015). However, molecular data relating to the period when a mesenchymal condensation becomes a complex bone structure are scarce. In the mouse, this occurs between day 13 and 15 of prenatal development.

Within these 2 days, avascular soft condensation turns into vascularized forming bone including osteoblasts, osteocytes and osteoclasts. We hypothesized that, during this important period, when osteogenesis reaches the point of all three major bone cell differentiation, vascularization already has to play a essential role. If this is the case, molecular changes supporting both types of cellular events, should take place. The purpose of this investigation therefore was to identify major osteogenic and angiogenic markers displaying expression alterations within this critical developmental period.

## Materials and Methods

### Animals

Mice (strain CD1) were purchased from the Breeding Units of Masaryk University Brno and kept in the facilities of the Institute of Animal Physiology and Genetics, Czech Academy of Sciences, Czechia. Mouse heads at stages between prenatal/embryonic (E) day 13–15 were used. Breeding of mice was performed in 2 h cycles to guarantee exact staging of the offspring. Pregnant mice were euthanized according to the experimental protocol approved by the Laboratory Animal Science Committee of the IAPG CAS, v.v.i., Brno, Czechia (project GA CR 17-14886S).

### Histological/Immunohistological Analysis of the Mandibular Bone

For histological and immunohistochemical analyses, mouse heads were fixed in 4% paraformaldehyde, dehydrated (ethanol series), treated with xylene, and embedded in paraffin.

Sections of heads in the region of the first mandibular molar segment (5 μm) were used for histological analysis (trichrome staining, von Kossa), immunohistochemistry (IHC) and detection of osteoclasts (TRAP assay). Histological sections were deparaffinised in xylene and rehydrated in a gradient series of ethanol, finishing in water.

For IHC, primary antibodies were applied as follows: CD31 (ab28364, Abcam; 1:100), osteopontin (ab91655, Abcam; 1:100), osteocalcin (ab93876, Abcam; 1:100), sclerostin (AF1589, R&D Systems; 1:200). ABC kit (Vectastain) was used for visualization of primary antibodies. Color reaction was achieved by chromogen POD-DAB.

Tartrate resistant acid phosphatase (TRAP) was detected using the Naphthol AS-TR phosphate disodium salt (0.0023M, N6125; Sigma-Aldrich, Germany), glacial acetic acid (0.2 M), sodium acetate (0.2 M), sodium tartrate dibasic dihydrate (0.1 M, S-8640; Sigma-Aldrich, Munich, Germany), N-N-dimethylformamide (0.5%) for 1 h at 37°C. Haematoxylin was used as counterstain.

### Tissue Separation

**FIGURE 1:**
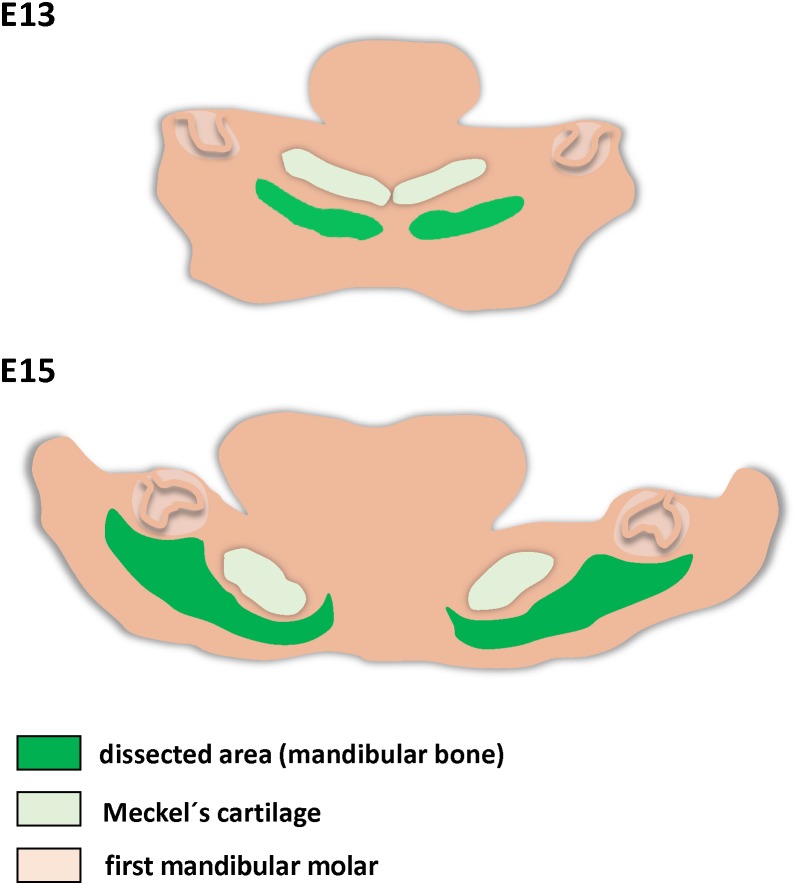
Schema of mandibular bone separation for PCR Array analysis. Dissected regions are green.

For PCR Array, fresh samples were microdissected as described previously (Minarikova et al., 2015). Briefly, mouse mandibles (E13 and E15) were separated and sliced into 250 μm thin slices by Mcllwain tissue chopper. Further, tissue slices with region of interest (Figure 1) were selected and the mandibular bone facing the first molar was segregated from surrounding tissue by needles using stereoscope. The samples were lysed by RLT buffer (Qiagen) for RNA isolation.

### PCR Array

RNeasy Kit (Qiagen) was used for RNA isolation, then mRNA was transcribed into cDNA using SuperScript VILO (Invitrogen), and PCR Arrays (Qiagen) were applied in osteogenic (Qiagen, PAMM-026Z) and angiogenic (Qiagen, PAMM-024Z) variants.

Data were statistically evaluated by Qiagen Data Analysis Center as recommended by manufacturer (available on-line). Statistical significance was determined as *p* < 0.05, the threshold of fold regulation as ±2. Three independent biological samples were analyzed for each stage. Genes included in PCR Array are listed in the Supplementary Material. Control housekeeping genes included: Actb, B2m, Gapdh, Gusb, and Hsp90ab1. The PCR Array format included positive and negative controls.

## Results

### Early Mandibular Bone Formation

**FIGURE 2:**
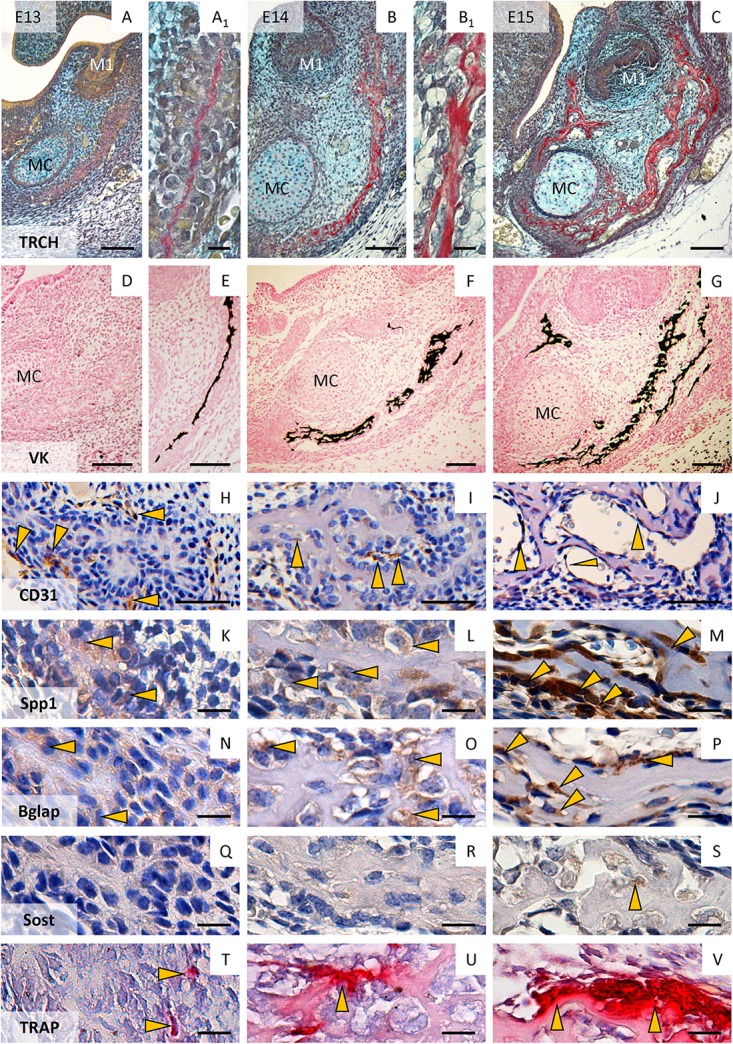
Formation of the mandibular bone in the region of the first lower molar at E13–E15. Morphology of the mandibular bone (trichrome staining, collagen is detected by Sirius red) at E13 **(A,A_1_)**, E14 **(B,B_1_)**, and E15 **(C)**; detection of mineralized tissue (von Kossa – mineralized parts are black) at E13 **(D)**, E13.5 **(E)**, E14 **(F)**, E15 **(G)**; immunohistochemical detection of endothelial cells (CD31) at E13 **(H)**, E14 **(I)**, E15 **(J)**; immunohistochemical localization of osteopontin (Spp1) at E13 **(K)**, E14 **(L)**, and E15 **(M)**; osteocalcin (Bglap) at E13 **(N)**, E14 **(O)**, E15 **(P)**; sclerostin at E13 **(Q)**, E14 **(R)**, and E15 **(S)**; detection of TRAP positive cells (pre-/osteoclasts) at E13 **(T)**, E14 **(U)**, E15 **(V)**. Arrows point to positive cells. M1, first molar; MC, Meckel’s cartilage. Scale bar **(A–G)** = 100 μm; **(H–J)** = 50 μm; (**A_1_,B_1_,K–V)** = 10 μm.

Early formation of mandibular bone in the segment connected with first molar tooth development starts as the condensation of mesenchymal cells located underneath the tooth germ, producing a thin layer of collagenous matrix (Figure 2A,A_1_). This became morphologically apparent at the prenatal/embryonic day (E)13. Mineralization was not visible (Figure 2D) at this time, however, it appeared a half day later (Figure 2E). CD31-positive endothelial cells were localized in surrounding bone (Figure 2H). The condensed mesenchymal cells were slightly positive for osteopontin (Figure 2K), osteocalcin (Figure 2N) and negative for sclerostin (Figure 2Q). Mononuclear TRAP-positive cells could be observed in bone proximity (Figure 2T).

One day later (E14) when the extracellular matrix (ECM) of the forming bone became more apparent (Figure 2B,B_1_) and mineralized (Figure 2F), CD31-positive endothelial cells invaded the mandibular bone (Figure 2I). Osteopontin (Figure 2L) and osteocalcin (Figure 2O) expression increased (compared with E13), sclerostin was rarely present (Figure 2R). Poly-nuclear TRAP-positive cells were detected adjacent to bone matrix (Figure 2U).

At E15, the mandibular bone synthesis (Figure 2C) and mineralization (Figure 2G) progressed, CD31-positive endothelial cells could be detected in vessels of mandibular bone (Figure 2J). Osteopontin (Figure 2M) and osteocalcin (Figure 2P) were strongly expressed, whereas the first sclerostin positive cells could be found at this stage (Figure 2S). Giant multinucleated osteoclasts appeared (TRAP-positive) on the margins of forming bone (Figure 2V).

### Osteogenic Profile of Cells Within the Forming Mandibular Bone

**FIGURE 3:**
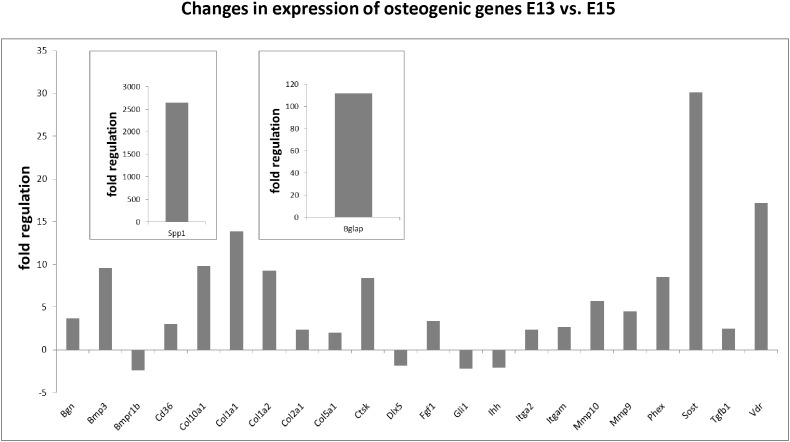
Changes in expression of osteogenic genes in developing mandibular bone (E13 vs. E15). Analysis shows fold regulations (–2/+2 is used as the threshold) of gene expression between E13–E15 detected by PCR Array. Statistically significant changes are displayed.

Using the osteogenic array, expression of 23 genes was found to be significantly up/downregulated between E13 and E15 in mandibular bone, with at least a twofold change. The most striking alterations were detected in osteopontin/Spp1 (2644-fold), osteocalcin/Bglap (112-fold), sclerostin/Sost (30-fold), vitamin D receptor/Vdr (17.17), Col1a1 (13.88), Col1a2 (9.29), cathepsin K/Ctsk (8.45) or phosphate regulating endopeptidase homolog X-linked/Phex (8.53). Complete list of variations in osteogenic gene expression is summarized in Figure 3. There were also genes with high and constant expression in both examined stages such as bone morphogenetic factors/Bmps, Smads, Runx2, or Nfkb1. The list of genes with high but constant expression between the investigated stages is provided in Table 1. Scheme of mandibular bone progression in context of osteogenic factors with detected altered expression between the investigated stages is shown in Figure 5.

**Table 1:** Osteogenic genes expressed at the level of housekeeping genes (Ct = 17–23) in both analyzed stages (E13 and E15) detected by PCR Arrays.

Constant expression of osteogenic genes (E13 vs. E15)			
Acvr1	Bmpr2	Itgav	Smad5^∗^
Alpl	Cdh11	Itgb1	Sox9
Anxa5	Col3a1	Mmp2^∗^	Sp7
Bmp1	Col4a1	Nfkb1	Tgfb3^∗^
Bmp2	Fgfr1	Runx2	Tgfbr1
Bmp4	Fgfr2	Serpinh1	Tgfbr2
Bmp5	Fn1^∗^	Smad1	Tnfsf11
Bmp6	Gdf10	Smad2	Twist1
Bmp7	Igf1^∗^	Smad3	Vegfa^∗^
Bmpr1a	Igfr1	Smad4	Vegfb


*Genes marked by asterisk are present in osteogenic and angiogenic panels.*

### Angiogenic Profile of Cells Within the Forming Mandibular Bone

**FIGURE 4:**
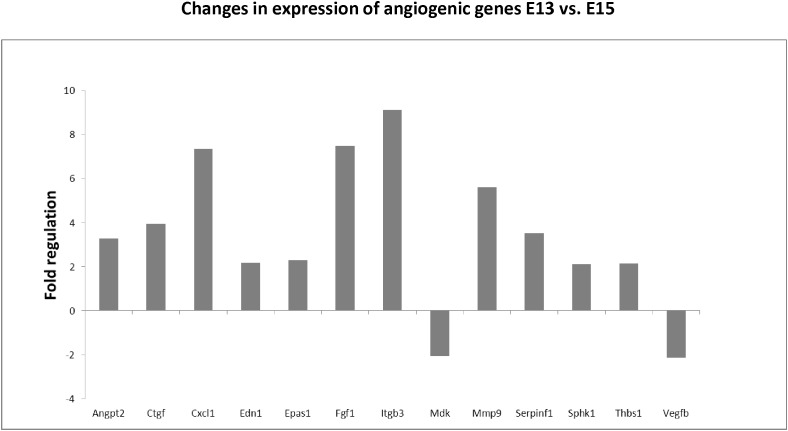
Changes in expression of angiogenic genes in developing mandibular bone (E13 vs. E15). Analysis shows fold regulations of gene expression (–2/+2 is used as the threshold) between E13–E15 detected by PCR Array. Statistically significant changes are displayed.

**FIGURE 5:**
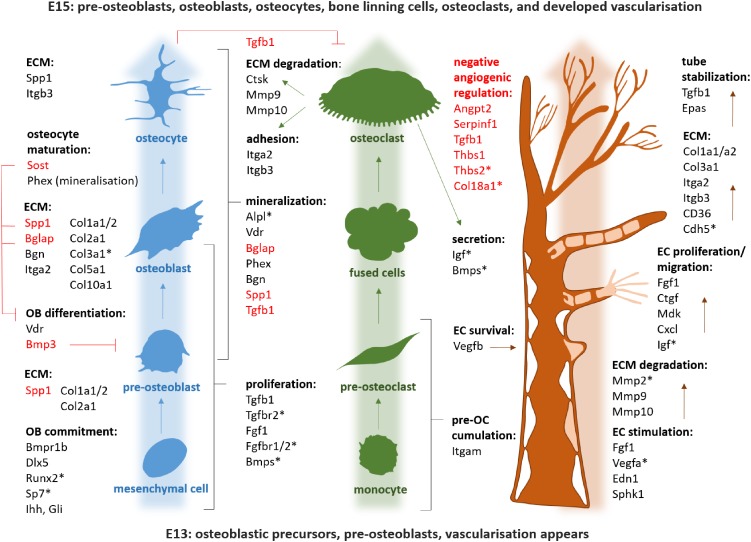
Scheme of the mandibular bone progression in context of osteogenic and angiogenic factors detected by PCR Array. Genes with high and stable expression all along the analyzed period are marked by asterisk. Negative regulators are red. OB, osteoblast; OC, osteoclasts; EC, endothelial cells; ECM, extracellular matrix.

Using the angiogenic array, 13 genes were found to be significantly up/downregulated between E13–E15 of mandibular bone development, with changes over twofold. The most prominent changes were observed in expression of integrin beta 3/Itgb3 (9.1-fold), fibroblast growth factor 1/Fgf1 (7.5-fold), chemokine ligand 1/Cxcl1 (7.4-fold), or matrix metalloproteinase 9/Mmp9 (5.6-fold). All alterations in angiogenic panel of genes are summarized in Figure 4. Factors with high and constant expression comprised transforming growth factors/Tgfbs, Pecam/CD31 or vascular endothelial growth factor/Vegfa. The list of genes with high but constant expression between the investigated stages is provided in Table 2. Scheme of mandibular bone progression in context of angiogenic factors with detected altered expression between the investigated stages is shown in Figure 5.

**Table 2:** Angiogenic genes expressed at the level of housekeeping genes (Ct = 17–23) in both analyzed stages (E13 and E15) detected by PCR Arrays.

Constant expression of angiogenic genes (E13 vs. E15)		
Akt1	Jag1	Smad5^∗^
Anpep	Kdr	Tgfb1
Cdh5	Mapk14	Tgfb2
Col18a1	Mmp14	Tgfb3^∗^
Efnb2	Mmp2^∗^	Tgfbr1^∗^
Eng	Nrp1	Thbs2
Fgfr3	Nrp2	Tie1
Fn1^∗^	Pecam1	Vegfa^∗^
Hif1a	Ptk2	
Igf1^∗^	S1pr1	


*Genes marked by asterisk are present in osteogenic and angiogenic panels.*

## Discussion

Given its relevance to craniofacial bone defect repair (Fishero et al., 2015; Tian et al., 2018) and dental implantology (Buser et al., 2017; Elgali et al., 2017), the mandibular bone is a common model in basic and clinical research. Most investigations into its formation have focused on the pre-condensation period (e.g., Jabalee et al., 2013; Kaul et al., 2015). When dealing with later stages related to cell differentiation, several *in vitro* approaches have been developed (e.g., Krishnan et al., 2010), although the physiological tissue/organ context was lost. For this purpose, direct evaluation of samples from *in vivo* developing bone where the complex set of cell interactions would be maintained was performed.

The transition period investigated here includes several successive and overlapping events: expansion of osteoblastic cells, maturation of osteoblasts, ECM deposition and mineralisation, osteocyte maturation, angiogenic progression, osteoclastic differentiation, and initial bone remodeling (Berendsen and Olsen, 2015). The dramatic morphological changes must be based on specific molecular signaling, which has not yet investigated with respect to the transition stages. Therefore the present study aimed to screen angiogenic and osteogenic expression profiles at the early post-condensation stages, to search for markers associated with the establishment of a complex vascularized bone. In agreement with our hypothesis suggesting the possibility of a synergic role of vascularization during the very initial steps of osteogenesis, the results pointed to significant expression alterations of 37 genes, out of 161 analyzed factors.

Within early osteoblastic inductors, our analysis showed a reduction in the expression of Bmpr1b and Dlx5 and also of components of the hedgehog pathway (Ihh, Gli) (Francis-West et al., 1994; Nie et al., 2005; Ulsamer et al., 2008). Simultaneously, the pattern of specific markers also paralleled the increased number of osteoblastic cells. Among these, Tgfb1 (Oka et al., 2007) and Fgf1 (Ornitz and Marie, 2015) along with their receptors (Tgfβr2 and Fgfr1/2) were elevated or strongly expressed during the examined period. Additionally, a significant increase was observed in the expression of Col1a1/2, which encodes the most abundant compound of the ECM (Matsuura et al., 2014). In the case of ECM mineralisation-related factors (e.g., Feng et al., 2013; Berendsen et al., 2014; Zvackova et al., 2017), biglycan expression was the most significantly increased during the formation of complex bone, and other molecules which showed increased expression included Vdr, Bglap, Phex, and Tgfb1.

As expected, the massive differentiation of osteoblasts during the examined period was associated with a huge increase in the levels of markers of osteoblastic differentiation (Zohar et al., 1998), Spp1 and Bglap, which were confirmed at the protein level by immunohistochemical analysis of mandibular bone. The Bmp pathway components (Chen et al., 2012) and Runx2 (Bruderer et al., 2014), which are fundamental for the subsequent steps of bone development, were maintained at consistently high levels of expression.

At day 15, the first osteocytes which displayed Sost-positivity, appeared in the mandibular bone region that was investigated by immunohistochemistry. These findings corresponded with the data at the transcriptome level. Among the integrins that are necessary for this period of development, increased levels of Itga2, Itgb3 (Haugh et al., 2015), Itgb3 and Itgam (Yang et al., 2017) were observed.

The appearance of integrins was associated also with osteoclasts (Nesbitt et al., 1993; Nakamura et al., 1999). The first TRAP-positive cells (Takeshita et al., 2000) adjacent to the developing mandibular bone were identified in the histological sections as early as day 13, however, they were still mononuclear. Two days later, these cells had acquired a giant volume and multicellular morphology (Alfaqeeh et al., 2013), and intense bone remodeling had been initiated to accommodate the growing tooth germ (Radlanski et al., 2015). In the case of proteases that are critical for bone resorption (Salo et al., 1997); Ctsk, Mmp9, and Mmp10 were upregulated during the investigated period. These molecules are necessary also for vascular ECM remodeling (Burbridge et al., 2002; Bruni-Cardoso et al., 2010).

The migration of osteoclastic precursors from the bone marrow is dependent on the vascular network. Vessels are visible around the mesenchymal condensation which precedes mandibular bone, as early as day 13. However, histological analysis revealed that these vessels penetrate the condensation a day later, and the bone structure was vascularized by day 15. In term of molecules involved in initiation and budding (Morbidelli et al., 1995; Xue and Greisler, 2002; Spiegel and Milstien, 2003); Fgf1, Edn1, and Sphk1 showed increased expression within the investigated period. Vascularisation is associated with hypoxia-inducible factors; in the present case, Hif2/Epas2 was increased. Among the molecules connected with vessel elongation (reviewed, e.g., in Rhodes and Simons, 2007); increased expression of Cd36, Itga2, Itgb3, and Col5a1 was observed, and constantly high expression of Col3a1, Col4a1, and Cdh5 were detected during the investigated period of mandibular bone development.

This report focused on a transient but very important period of mandibular bone formation, during which an osteogenic mesenchymal condensation becomes a complex bone structure. Although we did not aim to identify new genes, the results contribute to the limited knowledge which exists relating molecular factors to specific steps of osteogenesis and angiogenesis. The expression levels of 37; out of the 161 analyzed; osteogenic and angiogenic factors, were altered compared between the condensation stage and bone stages, which strongly support our hypothesis also concerning the relationship between vascularization and the very early stages of osteogenesis. Expression profile data that is specific for a particular period of development, such as that presented here, provides an overview of the molecular network that is active in the post-condensation stage prior to the appearance of complex bone, and can offer an important foundation for further experimental studies.

## Author Contributions

BV performed the sample preparation, PCR Arrays and following analysis, and contributed to manuscript preparation. EŠ performed the tissue dissection, (immuno) histological staining, and contributed to manuscript construction. JB contributed to preliminary data and participated in manuscript preparation. HL contributed to data analysis and manuscript preparation. EM was head of this project, contributed to the research design, and manuscript construction and completion.

## Conflict of Interest Statement

The authors declare that the research was conducted in the absence of any commercial or financial relationships that could be construed as a potential conflict of interest.
