# Supplementary material for: Osteogenic and Angiogenic Profiles of Mandibular Bone-Forming Cells
Source: Front Physiol. 2019 Feb 19;10:124. doi: 10.3389/fphys.2019.00124 (PMC6389724; doi:10.3389/fphys.2019.00124)
Supplement: Supplementary file 1 [file Table_1.DOCX]

**Supplement 1**

**Osteogenic PCR Array**

The Mouse Osteogenesis RT² Profiler™ PCR Array (Qiagen, PAMM-026Z) gene list:

Acvr1 (Activin A receptor, type I ), Ahsg (Alpha 2-HS Glycoprotein ), Alpl (Alkaline Phosphatase, Liver/Bone/Kidney), Anxa5 (Annexin A5), Bglap (Osteocalcin), Bgn (Biglycan), Bmp1 (Bone Morphogenetic Protein 1), Bmp2, Bmp3, Bmp3b/Gdf10 (Growth Differentiation Factor 10 ), Bmp4, Bmp5, Bmp6, Bmp7, Bmpr1a (Bone Morphogenetic Protein Receptor Type 1A), Bmpr1b, Bmpr2, Cd36 (Thrombospondin Receptor), Cdh11 (Cadherin 11), Chrd (Chordin), Col1a1 (Collagen Type I Alpha 1 Chain), Col1a2, Col2a1, Col3a1, Col4a1, Col5a1, Col10a1, Col14a1, Comp (Cartilage Oligomeric Matrix Protein), Csf1/Mcsf (Colony Stimulating Factor 1/ Macrophage Colony Stimulating Factor 1), Csf2/GM-CSF (Colony Stimulating Factor 2), Csf3/GCSF (Colony Stimulating Factor 3), Ctsk (Cathepsin K), Dlx5 (Distal-Less Homeobox 5), Egf (Epidermal Growth Factor), Fgf1 (Fibroblast Growth Factor 1), Fgf2/bFGF, Fgfr1 (Fibroblast Growth Factor Receptor 1), Fgfr2, Flt1 (Fms Related Tyrosine Kinase 1), Fn1 (Fibronectin 1), Gli1 (GLI Family Zinc Finger 1 ), Igf1 (Insulin Like Growth Factor 1), Igf1r (Insulin Like Growth Factor 1 Receptor ), Ihh (Indian Hedgehog), Icam1 (Intercellular Adhesion Molecule 1), Itga2 (Integrin Subunit Alpha 2), Itga2b (Integrin Subunit Alpha 2b), Itga3, Itgam (Integrin Subunit Alpha M), Itgav (Integrin Subunit Alpha V), Itgb1 (Integrin Subunit Beta 1), Mmp2 (Matrix Metallopeptidase 2), Mmp8, Mmp9, Mmp10, Nfkb1 (Nuclear Factor Kappa B Subunit 1), Nog (Noggin), Pdgfa (Platelet Derived Growth Factor Subunit A), Phex (Phosphate Regulating Endopeptidase Homolog X-Linked), Runx2 (Runt Related Transcription Factor2),Serpinh1 (Serpin Family H Member 1), Smad1 (SMAD Family Member 1), Smad2, Smad3, Smad4, Smad5, Sost (Sclerostin), Sox9 (SRY-Box 9), Sp7 (Osterix), Spp1 (Osteopontin), Tgfb1 (Transforming Growth Factor Beta 1), Tgfb2, Tgfb3, Tgfbr1 (Transforming Growth Factor Beta Receptor 1), Tgfbr2, Tgfbr3, Tnf (Tumor Necrosis Factor), Tnfsf11 (TNF Superfamily Member 11), Twist1 (Twist Family BHLH Transcription Factor 1), Vcam1 (Vascular Cell Adhesion Molecule 1), Vdr (Vitamin D Receptor), Vegfa (Vascular Endothelial Growth Factor A), Vegfb.

**Angiogenic PCR Array**

The Mouse Angiogenesis RT² Profiler™ PCR Array (Qiagen, PAMM-024Z) gene list:

Angpt1 (Angiopoietin 1), Angpt2, Anpep (Alanyl Aminopeptidase, Membrane), Bai1 (Adhesion G Protein-Coupled Receptor B1), Ccl11 (C-C Motif Chemokine Ligand 11), Ccl2, Cdh5 (Cadherin 5), Col4a3 (Collagen Type IV Alpha 3 Chain), Col18a1, Csf3 (Colony Stimulating Factor 3), Ctgf (Connective Tissue Growth Factor ), Cxcl1 (C-X-C Motif Chemokine Ligand 1), Cxcl2, Cxcl5, Efna1 (Ephrin A1), Efnb2, Egf (Epidermal Growth Factor), Eng (Endoglin), Epas1/Hif2a (Endothelial PAS Domain Protein 1), Ephb4 (EPH Receptor B4 ), Ereg (Epiregulin), F2/CF-2 (Coagulation Factor II, Thrombin), Fgf1 (Fibroblast growth factor 1), Fgf2, Fgf6, Fgfr3, Flt1 (Fms Related Tyrosine Kinase 1), Fzd5 (Frizzled Class Receptor 5), Gna13 (G Protein Subunit Alpha 13), Hand2 (Heart And Neural Crest Derivatives Expressed 2), Hgf (Hepatocyte Growth Factor), Hif1a (Hypoxia Inducible Factor 1 Alpha Subunit), Ifng (Interferon Gamma), Igf1 (Insulin Like Growth Factor 1), Il1b (Interleukin 1 Beta), Il6 (Interleukin 6), Itgb3 (Integrin Subunit Beta 3), Itgav (Integrin Subunit Alpha V), Jag1 (Jagged 1), Kdr Kinase Insert Domain Receptor), Lama5 (Laminin Subunit Alpha 5), Lect1 (Chondromodulin), Lep (Leptin), Mapk14 (Mitogen-Activated Protein Kinase 14), Mdk (Midkine), Mmp2 (Matrix Metallopeptidase 2), Mmp9, Mmp19, Nrp1 (Neuropilin 1), Nrp2, Pdgfa (Platelet Derived Growth Factor Subunit A), Pecam1 (Platelet Derived Growth Factor Subunit A), Pgf (Placental Growth Factor), Plau (Plasminogen Activator, Urokinase), Plg (Plasminogen), Plxdc1 (Plexin Domain Containing 1), Ptgs1 (Prostaglandin-Endoperoxide Synthase 1), S1pr1 (Sphingosine-1-Phosphate Receptor 1), Serpinf1 (Serpin Family F Member 1), Smad5 (SMAD Family Member 5), Sphk1 (Sphingosine Kinase 1), Stab1 (Stabilin 1), Tbx1 (T-Box 1), Tbx4, Tgfa (Transforming Growth Factor Alpha), Tgfb1, Tgfb2, Tgfb3, Tgfbr1 (Transforming Growth Factor Beta Receptor 1), Tek (TEK Receptor Tyrosine Kinase), Timp1 (TIMP Metallopeptidase Inhibitor 1), Timp2, Thbs1 (Thrombospondin 1), Thbs2, Tmprss6 (Transmembrane Protease, Serine 6), Tnf (Tumor Necrosis Factor), Tnfaip2 (TNF Alpha Induced Protein 2), Tnfsf12 (TNF Superfamily Member 12), Tymp (Thymidine Phosphorylase), Vegfa (Vascular Endothelial Growth Factor A), Vegfb, Vegfc, Vegf-d/Figf.
